# HIV Drug Resistance and Transmission Networks Among a Justice-Involved Population at the Time of Community Reentry in Washington, D.C.

**DOI:** 10.1089/aid.2020.0267

**Published:** 2021-12-15

**Authors:** Curt G. Beckwith, Sugi Min, Akarsh Manne, Vladimir Novitsky, Mark Howison, Tao Liu, Irene Kuo, Ann Kurth, Lauri Bazerman, Anya Agopian, Rami Kantor

**Affiliations:** ^1^ Division of Infectious Diseases, The Miriam Hospital, Providence, Rhode Island, USA.; ^2^ The Warren Alpert Medical School of Brown University, Providence, Rhode Island, USA.; ^3^ Research Improving People's Lives, Providence, Rhode Island, USA.; ^4^ Department of Biostatistics, Brown University School of Public Health, Providence, Rhode Island, USA.; ^5^ George Washington University Milken Institute School of Public Health, Washington, District of Columbia, USA.; ^6^ Yale University School of Nursing, Orange, Connecticut, USA.

**Keywords:** HIV, drug resistance, transmission networks, justice populations

## Abstract

Justice-involved (JI) populations bear a disproportionate burden of HIV infection and are at risk of poor treatment outcomes. Drug resistance prevalence and emergence, and phylogenetic inference of transmission networks, understudied in vulnerable JI populations, can inform care and prevention interventions, particularly around the critical community reentry period. We analyzed banked blood specimens from CARE+ Corrections study participants in Washington, D.C. (DC) across three time points and conducted HIV drug resistance testing using next-generation sequencing (NGS) at 20% and 5% thresholds to identify prevalent and evolving resistance during community reentry. Phylogenetic analysis was used to identify molecular clusters within participants, and in an extended analysis between participants and publicly available DC sequences. HIV sequence data from 54 participants (99 specimens) were analyzed. The prevalence of transmitted drug resistance was 14% at both thresholds, and acquired drug resistance was 47% at 20%, and 57% at 5% NGS thresholds, respectively. The overall prevalence of drug resistance was 43% at 20%, and 52% at 5% NGS thresholds, respectively. Among 34 participants sampled longitudinally, 21%–35% accumulated 10–17 new resistance mutations during a mean 4.3 months. In phylogenetic analysis within the JI population, 11% were found in three molecular clusters. The extended phylogenetic analysis identified 46% of participants in 22 clusters, of which 21 also included publicly-available DC sequences, and one JI-only unique dyad. This is the first study to identify a high prevalence of HIV drug resistance and its accumulation in a JI population during community reentry and suggests phylogenetic integration of this population into the non-JI DC HIV community. These data support the need for new, effective, and timely interventions to improve HIV treatment during this vulnerable period, and for JI populations to be included in broader surveillance and prevention efforts.

## Introduction

The justice-involved (JI) population bears a disproportionate burden of HIV infection in the United States, with an estimated one-in-seven persons with HIV passing through correctional facilities each year.^1^ While antiretroviral therapy (ART) adherence during incarceration is generally high, challenges surrounding release from a correctional facility, known as “community reentry,” can lead to ART interruption, relapse to substance use, poor adherence,^2^ and viral rebound,^3^ all of which can contribute to the development of drug resistance mutations (DRMs) that can be transmitted to sexual and drug-using partners. Development and transmission of ART resistance pose significant threats to effective, long-term virologic suppression among this high-risk population.

Despite the increased risk of drug resistance among JI persons with HIV, its prevalence and emergence in this population, particularly during the time of community reentry, remain understudied. To date, only four U.S. studies were conducted among JI populations, at various time points in the HIV epidemic, demonstrating a wide range (16%–43%) of drug resistance.^4–7^ However, all these studies focused on incarcerated individuals, similar to such studies performed outside of the United States.^8–11^ To our knowledge, there have been no studies that have examined the emergence of drug resistance among persons with HIV recently released from correctional facilities.

HIV sequence data obtained for resistance testing can also be used for phylogenetic inference of transmission networks to identify key epidemiological risk factors driving transmission and to guide prevention efforts in real time.^12–14^ However, such studies have rarely been conducted among JI populations. One U.S. study in Georgia identified 88 persons with HIV seroconversion while in prison, with 49% of the 67 viral sequences falling into 1 of 10 genetically related clusters.^7^ The majority (76%) of persons with genetically related HIV strains reported sex in prison before their diagnosis, providing support for the presence of transmission networks within prison.^7^ In two Brazilian prisons, Cardoso *et al.* found three possible dyad clusters of intra-prison transmission among 27 inmates, while Prellwitz *et al.* found no transmission clusters among sequences from 40 inmates.^9,10^ Among phylogenetic studies using larger, regional-level databases, incarceration history is not used explicitly as a risk factor for HIV transmission.

Given that the community reentry period presents challenges that can impact ART adherence and linkage to care, and can be associated with viral rebound, we examined whether this critical period contributed to the emergence of drug resistance in a JI population in Washington, D.C. (DC). We also conducted phylogenetic analyses with inclusion of this JI population and hypothesized that it is part of networks among the greater DC community, knowledge that could inform local transmission prevention strategies. DC is an important setting for this study, given that its HIV prevalence parallels some sub-Saharan African nations^15^ and it was recently designated as a geographic high-priority hotspot for community intervention as part of the national “Ending the HIV Epidemic” initiative.^16^

## Materials and Methods

### Parent study population

Participants were originally enrolled in the CARE+ Corrections study between August 24, 2013 and April 30, 2015, which evaluated a mobile health intervention to improve HIV treatment and viral suppression in DC among 110 persons with HIV released from correctional facilities.^17^ Eligibility criteria included living with HIV, age ≥18 years, incarcerated in the DC Department of Corrections with an anticipated release date of ≤6 weeks, or residing in the community but having been released from a jail, prison, or halfway house within the previous 6 months. Participants completed a baseline assessment that included demographic characteristics, justice history, HIV care engagement, ART adherence, sexual and substance use behaviors, and mental health and other comorbid conditions. Prior 30-day ART adherence was assessed using a visual analog scale (0%–100%).^18^

Blood specimens were obtained to measure baseline HIV-1 plasma viral load (PVL) and CD4 cell counts, and participants were seen at 3 and 6 months for repeat PVL testing and ART adherence assessments. Specimens were sent to Miriam Hospital in Providence, Rhode Island for HIV PVL testing conducted by the Roche Cobas AmpliPrep/Cobas Taqman HIV-1 Test, Version 2.0 (Roche Diagnostics, Indianapolis, IN) with a lower limit of detection of 20 copies/mL. CD4 cell count testing was completed by a commercial lab (LabCorp) via the Becton Dickinson Canto II flow cytometer (Franklin Lakes, NJ). When study staff were unable to obtain blood from venipuncture or if a participant did not complete PVL and/or CD4 count testing during the baseline visit, Department of Corrections or community medical records from the 6-month period before the baseline assessment were reviewed to collect PVL and CD4 count data to establish baseline values.

### Current study population

Baseline (enrollment), 3-month, and 6-month specimens were banked for future testing (including drug resistance) with participants' consent. The study population included 55/110 participants with detectable PVL (>200 copies/mL) at any time point. Recent ART history was obtained from medical records at study enrollment. The CARE+ Corrections study was approved by the Institutional Review Boards at George Washington University and The Miriam Hospital in Providence, RI.

### Drug resistance testing and analysis

Drug resistance genotypic testing was attempted for samples with PVL >200 copies/mL using polymerase chain reaction amplification and next-generation sequencing (NGS) of the protease (HXB2 nucleotide positions 2253–2558), reverse transcriptase (RT; positions 2559–3869), and integrase (IN; positions 4230–5090) genes by Illumina MiSeq with Nextera^®^XT chemistry (Illumina, San Diego, CA). Sequence analysis and quality control were performed with HIVMMER.^19^ We used mafft to generate a multiple sequence alignment from the HIVMMER consensus sequences for each participant.^20^ Stanford Database tools^21^ were used for subtyping and for resistance interpretation at two NGS thresholds: 20%, equivalent to commonly used Sanger Sequencing^22^; and 5%, increasingly explored for clinically relevant minority resistance variants detection.^23^ Transmitted drug resistance (TDR) was determined among participants who were treatment-naïve at baseline based on (1) self-report, and (2) medical record review, and defined as the presence of at least one surveillance DRM included in the WHO Surveillance DRMs list^24,25^ in their earliest-available sequence. Acquired drug resistance (ADR) was determined among participants who were treatment-experienced at baseline, and defined as the presence of any DRM from the Stanford Database mutation list at any available sequence.^21^ The overall prevalence of drug resistance for the entire study population was determined by the sum of TDR and ADR.

For participants with multiple genotypes (at two or three time points), resistance accumulation was defined as a DRM from the Stanford Database mutation list detected at ≥5% NGS threshold in any follow-up specimen that was not detected in an earlier specimen. Mean number of DRMs for this group was compared between earliest and last available specimens and analyzed by Wilcoxon's signed-rank test. Accumulation rate for the study population was calculated as the total number of accumulated DRMs, divided by the person-months of follow-up between the earliest and last available specimens. Associations between the presence of DRMs at any time point and the accumulation of DRMs with available demographic and clinical characteristics at enrollment were analyzed by Student's *t*, rank-sum, chi-square, or exact tests.

### Phylogenetic analysis

**FIG. 4.**
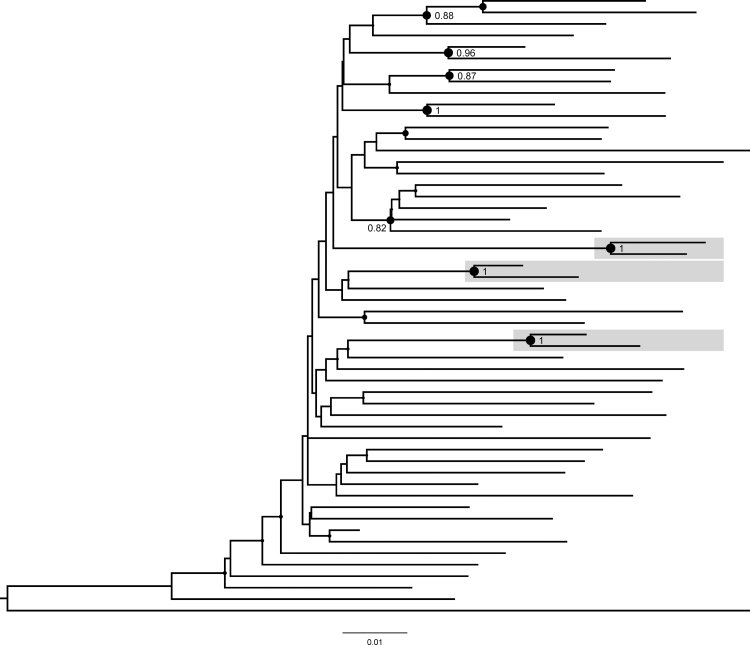
Maximum likelihood phylogenetic relationships among 54 study participants. The figure demonstrates a maximum likelihood phylogenetic sub-tree of the 54 study participants (see text for specific methods). Three identified clusters (all dyads) are highlighted in *gray boxes*. Bootstrap support ≥0.80 is shown at the node of each cluster and node size is proportional to bootstrap support. Other than one HIV-1 subtype C sequence (the *bottom* sequence on the tree), all were HIV-1 subtype B (see Materials and Methods section for HIV-1 subtyping details). The HIV-1 sequences that were used for tree rooting (three HIV-1 subtype G sequences, accession numbers BE.96.DRCBL.AF084936, KE.93.HH8793-12-1.AF061641, and NG.92.92NG083.U88826; and three HIV-1 subtype H sequences, accession numbers BE.93.VI991.AF190127, BE.93.VI997.AF190128, and CF.90.056.AF005496) are not shown. The tree scale is shown at the *bottom* of the figure.

A phylogeny was inferred from the multiple *pol* sequence alignment by maximum likelihood using RAxML^26^ with GTRCAT model and by the Neighbor-Joining method using uncorrected p-distances. Both trees were rooted using three HIV-1 subtype G and three HIV-1 subtype H sequences (see Fig. 4 legend for more details). A molecular HIV cluster was defined as a monophyletic subtree using one of two stringency levels: (1) a conventional strict definition increasingly used for outbreak investigations: bootstrap support ≥0.95 (1,000 replicates) and mean pairwise distances ≤0.015 substitutions/site; and (2) a relaxed definition, to explore historical, non-outbreak-related clustering: bootstrap support ≥0.80 (1,000 replicates) and mean pairwise distances ≤0.030 substitutions/site. These stringency levels were chosen to represent a spectrum of analytic options that are commonly used in the literature.^27^ We note that the commonly used nomenclature of “transmission networks” or clusters used throughout the article does not imply any linkage between members of such networks or clusters. Phylogeny was inferred using a single (earliest) sequence per participant. In an extended analysis, we combined participant sequences with publicly available 3,598 North American HIV-1 subtype B sequences from the Los Alamos National Lab HIV database (https://www.hiv.lanl.gov/content/index), including 1,812 DC sequences. Duplications were excluded by using the “One sequence/patient” option at the Los Alamos National Lab HIV database.

## Results

### Baseline characteristics

**FIG. 1.**
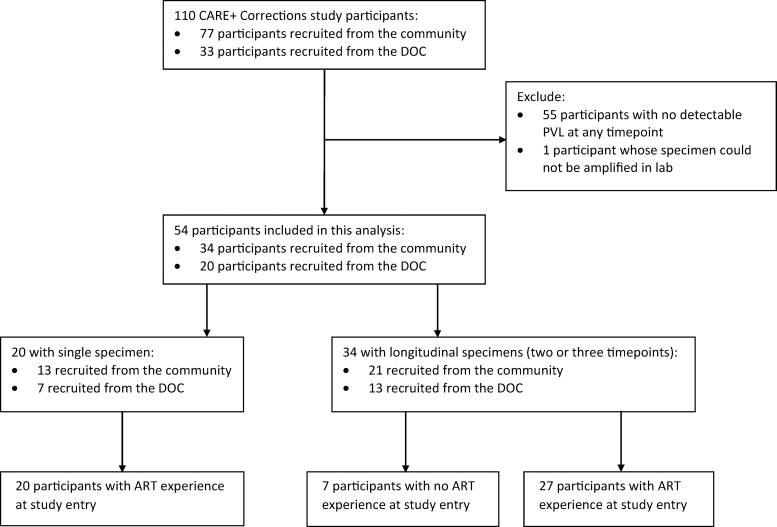
Flowchart of participants included in this analysis from the CARE+ Corrections parent study. ART, antiretroviral therapy; DOC, department of corrections; PVL, plasma viral load.

Figure 1 outlines the study population. Of the 55 of 110 participants with detectable PVL at any time point, genotypes were available for 54 participants and were included in this analysis. Table 1 includes baseline characteristics of the 54 participants, according to the place of enrollment. Notably, 59% (32/54) were male, 30% (16/54) female, and 11% (6/54) male-to-female transgender; median age was 38 years; 78% (42/54) were non-Hispanic black; median number of lifetime incarcerations was 7; HIV was diagnosed ≥5 years prior in 70% (38/54); 87% (47/54) were ART-experienced; and 52% (28/54) were on ART at enrollment, of whom 61% (17/28) reported ≥90% ART adherence before their most recent incarceration.

**Table 1.** Baseline Characteristics of the Study Participants with Detectable Viremia at ≥1 Time Point

Variable	Community (*n* = 34)	DOC (*n* = 20)	All (*n* = 54)	p
Study arm				.25
Control	17 (50%)	6 (30%)	23 (42.59%)	
Intervention	17 (50%)	14 (70%)	31 (57.41%)	
Age (years), median (IQR)	40.5 (30, 50)	36.5 (29.5, 48)	38.5 (30, 50)	.774 (rank-sum)
Identified gender				.244 (exact)
Male	17 (50%)	15 (75%)	32 (59.26%)	
Female	12 (35.29%)	4 (20%)	16 (29.63%)	
Transgender (MTF)	5 (14.71%)	1 (5%)	6 (11.11%)	
Race				.692 (exact)
Non-Hispanic white	2 (5.88%)	2 (10%)	4 (7.41%)	
Non-Hispanic black	26 (76.47%)	16 (80%)	42 (77.78%)	
Hispanic and others	6 (17.65%)	2 (10%)	8 (14.81%)	
Sexual orientation				.291 (exact)
Heterosexual	26 (76.47%)	18 (90%)	44 (81.48%)	
Lesbian, gay, or bisexual	8 (23.53%)	2 (10%)	10 (18.52%)	
Number of times incarcerated				.28 (exact)
≤5	13 (38.24%)	9 (45%)	22 (40.74%)	
>5	21 (61.76%)	11 (55%)	32 (59.3%)	
Length of incarceration, lifetime				.757
≤2 years	11 (32.35%)	5 (25%)	16 (29.63%)	
>2 years	23 (67.65%)	15 (75%)	38 (70.4%)	
Any mental health diagnosis	30 (88.24%)	14 (70%)	44 (81.48%)	.147 (exact)
Unstable housing	25 (73.53%)	16 (80%)	41 (75.93%)	.746 (exact)
Drug dependence^a^	25 (73.53%)	9 (45%)	34 (62.96%)	.0711
Ever injected drugs	7 (20.59%)	1 (5%)	8 (14.81%)	.234 (exact)
Recent noninjection drug use	24 (70.59%)	12 (60%)	36 (66.67%)	.618
Time since HIV diagnosis				.793
<5 years	11 (32.35%)	5 (25%)	16 (29.63%)	
≥5 years	23 (67.65%)	15 (75%)	38 (70.37%)	
Ever treated for HIV^b^	30 (88.24%)	17 (85%)	47 (87.04%)	1 (exact)
CD4 count, median (IQR)	359 (228, 634)	444 (268, 520)	432 (232, 578)	.790 (rank-sum)
Treated for HIV at study entry				.0747
No	8 (23.53%)	10 (50%)	18 (33.33%)	
Unknown	5 (14.71%)	3 (15%)	8 (14.81%)	
Yes	21 (61.76%)	7 (35%)	28 (51.85%)	
ART combination^c^				
NRTI+NNRTI	5 (23.81%)	4 (57.14%)	9 (32.14%)	
NRTI+PI	5 (23.81%)	3 (42.86%)	8 (28.57%)	
NRTI+InSTI^d^	5 (23.81%)	0 (0%)	5 (17.86%)	
NRTI+NNRTI+PI	1 (4.76%)	0 (0%)	1 (3.57%)	
NRTI+PI+InSTI^e^	3 (14.29%)	0 (0%)	3 (10.71%)	
Unknown	2 (9.52%)	0 (0%)	2 (7.14%)	
Preincarceration ART adherence >90%^f^				0.668 (exact)
No	12 (57.14%)	5 (71.43%)	17 (60.71%)	
Yes	9 (42.86%)	2 (28.57%)	11 (39.29%)	
Unknown	13	13	26	

^a^ TCU drug dependence scale.^28^

^b^ One participant indicated no ART history at baseline questioning but ART history was found on medical record review, therefore, participant was added to “Ever Treated for HIV” group.

^c^ ART regimen from chart review, percentages calculated among those reporting HIV treatment at study entry.

^d^ Specifically, InSTI use consisted of four patients on elvitegravir and one patient on raltegravir.

^e^ All three patients on raltegravir.

^f^ ART adherence measured with VAS.^17^

ART, antiretroviral therapy; DOC, department of corrections; InSTI, integrase strand transfer inhibitor; IQR, interquartile range; MTF, male-to-female; NNRTI, non-nucleoside reverse transcriptase inhibitor; NRTI, nucleoside reverse transcriptase inhibitor; PI, protease inhibitor; TCU, Texas Christian University; VAS, visual analog scale.

Genotyping was performed on 99 available specimens from the 54 participants: 31, 33, and 35 specimens from baseline, 3-month [range 2.2–5.1 months; interquartile range (IQR) 3.0–3.2], and 6-month (range 4.5–7.4 months; IQR 5.9–6.2) visits, respectively. For these groups of specimens, median PVL was 3.9 log10 copies/mL (range 2.4–5.7 log10 copies/mL), 3.9 log10 copies/mL (range 2.4–5.8 copies/mL), and 4.0 log10 copies/mL (range 2.3–5.6 copies/mL), respectively. The mean CD4 count at baseline was 432 cells/mm^3^ (IQR 232–578).

### Drug resistance prevalence

**FIG. 2.**
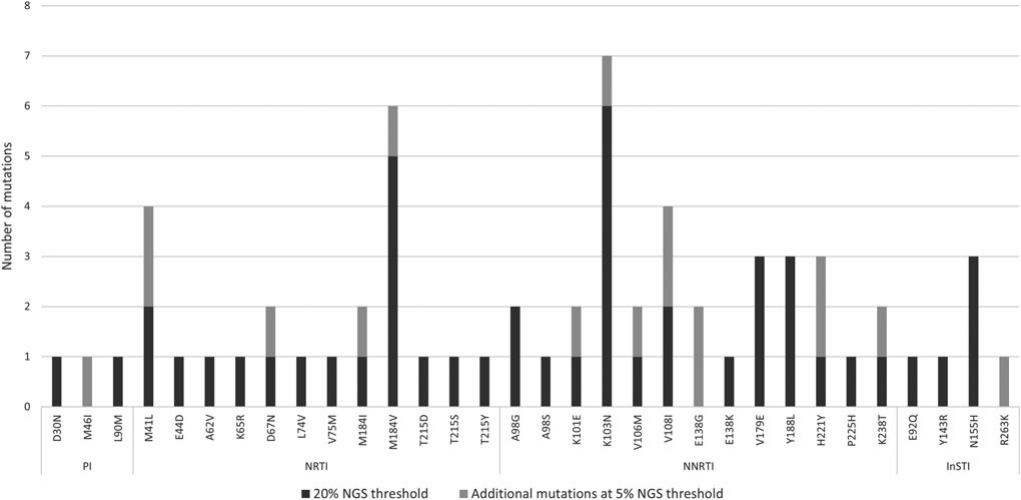
Prevalence of acquired DRMs by ART class among treatment-experienced participants (*n* = 47) at 20% and 5% NGS thresholds. DRM, drug resistance mutation; InSTI, integrase strand transfer inhibitor; NGS, next-generation sequencing; NNRTI, non-nucleoside reverse transcriptase inhibitor; NRTI, nucleoside reverse transcriptase inhibitor; PI, protease inhibitor.

TDR was found among 14% (1/7) of ART-naïve participants at both 20% and 5% thresholds (one protease inhibitor (PI)-associated L90M DRM). ADR was found among 47% (22/47) ART-experienced participants at 20% threshold [28% (13/47) nucleoside reverse transcriptase inhibitor (NRTI), 32% (15/47) non-nucleoside reverse transcriptase inhibitor (NNRTI), 4% (2/47) PI, 9% (4/47) integrase strand transfer inhibitor (InSTI)], and 57% (27/47) at 5% threshold [32% (15/47) NRTI, 36% (17/47) NNRTI, 6% (3/47) PI, 11% (5/47) InSTI]. Figure 2 lists the specific DRMs that contributed to ADR according to ART class. Combined among all 54 participants, the overall prevalence of drug resistance was 43% (23/54) at 20% NGS threshold [24% (13/54) NRTI, 31% (17/54) NNRTI, 6% (3/54) PI, 9% (5/54) InSTI], and 52% (28/54) at 5% threshold [28% (15/54) NRTI, 31% (17/54) NNRTI, 7% (4/54) PI, 9% (5/54) InSTI). There were no associations between demographic or clinical characteristics and the overall prevalence of DRMs.

### DRMs accumulation

**FIG. 3.**
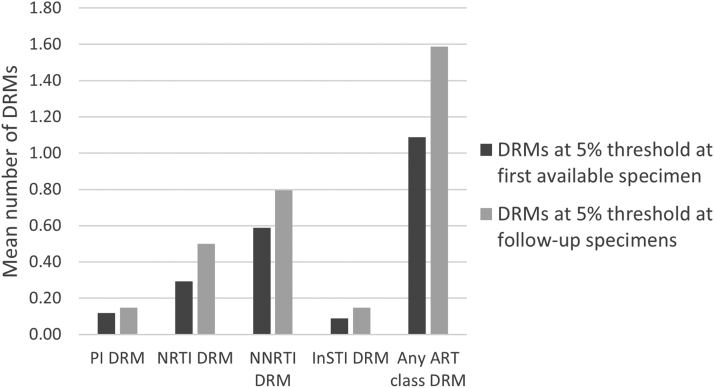
Accumulation of DRMs. Mean number of accumulated DRMs per person between first available specimen and follow-up specimens at 5% NGS threshold. ART, antiretroviral therapy.

DRM accumulation was analyzed for the 34 participants with longitudinal specimens (two or three timepoints), with a mean follow-up time of 4.3 months (IQR: 3.0–6.0 months) between first and last available specimens (Fig. 3). Among these 34 participants, 12 (35%) accumulated a combined 17 new DRMs, detected at the 5% threshold at follow-up time points but not present in the first available sample (Table 2). At the 20% threshold, 7/34 (21%) accumulated 10 DRMs. Overall, the mean DRM number in any ART class increased from 1.09 (range 0–11) at first-available sample to 1.59 (range 0–13) in follow-up samples (*p* = .0024) over mean 4.3 months; an overall DRM accumulation rate of 0.12 new DRMs per person-months at the 5% threshold (0.07 at the 20% threshold). The highest accumulation rates were seen for NRTI and NNRTI-associated mutations (0.05 per person-months). There were no associations between accumulated DRMs and demographic or clinical baseline characteristics.

**Table 2.** Drug Resistance Mutations Among 12 Participants with Longitudinal Samples and Accumulation of Drug Resistance Mutations at >5% Next-Generation Sequencing Threshold

Participant	Months between earliest and last specimen	ART regimen at baseline	PI DRM	NRTI DRM	NNRTI DRM	INSTI DRM
1	4.9	3TC/ABC/DRV/r		**M184I (38%), M184V (54%)**		
2	2.2	FTC/TDF/EFV			**K103N (22%), V106M (5%)**, V108I	
3	6.0	FTC/TDF/ELV/COBI	L90M	M41L, **K65R (24%)**, M184V, T215Y	A98G, **A98S (76%)**, V108I, V179E, Y188L, H221Y	Y143R, N155H
4	2.9	FTC/TDF/ELV/COBI			**V106M (100%)**	
5	6.9	FTC/TDF/ELV/COBI			**K238T (6%)**	
6	6.2	FTC/TDF/RAL		**M41L (10%), D67N (30%)**, M184V		N155H
7	3.2	No ART at enroll			K103N, **E138G (15%)**	
8	6.0	No ART at enroll		**T215S (6%)**		
9	6.1	No ART at enroll	**M46I (7%)**		V179D	
10	6.1	No ART at enroll				**R263K (11%)**
11	2.2	Unknown				**Y143H (30%)**
12	3.0	Unknown		**T215D (98%)**	**Y188L (98%)**	

Bold indicates mutations detected at ≥5% NGS threshold in any follow-up specimen that was not detected at the earliest available specimen; Percentages represent proportions of the viral quasispecies for accumulated mutations.

3TC, lamivudine; ABC, abacavir; ATV, atazanavir; COBI, cobicistat; DRV, darunavir; EFV, efavirenz; ELV, elvitegravir; FTC, emtricitabine; r, ritonavir; RAL, raltegravir; TDF, tenofovir.

### Phylogenetic analysis

Figure 4 demonstrates phylogenetic relationships of the 54 participants; 53 had HIV-1 subtype B and one subtype C. At the common strict threshold, no clusters were identified as all four dyads seen at the bootstrap support of ≥0.95 had pairwise distances >0.015 substitutions per site. At the exploratory relaxed threshold, 6/54 (11%) participants were found in 3 molecular clusters (all dyads). DRMs were not shared by cluster members. The branch topologies and identified clusters between the maximum likelihood (Fig. 4) and neighbor-joining trees (Supplementary Fig. S1) are similar.

In the extended phylogenetic analysis including 3,598 publicly available sequences from North America (2011–2016; including 1,812 from DC) and using stricter phylogenetic cluster definition criteria, 15 (28%) participants were found in 14 clusters with other DC sequences, including one triad with two JI cohort participants (who formed a dyad in the JI-cohort-only analysis with strong bootstrap support of 100% and pairwise distance 1.9%). Using the exploratory relaxed threshold, 25 (46%) participants were found in 22 clusters including 21 clusters with other, non-justice DC sequences, and a dyad cluster unique to the JI cohort (the same cluster was seen in the JI cohort-only analysis with strong bootstrap support of 100% and pairwise distance 1.5%).

## Discussion

JI persons with HIV are at high risk of poor HIV outcomes, and community reentry is an especially high-risk period associated with challenges that can lead to treatment interruption, poor adherence, and virological failure.^2,3^ We demonstrated that HIV drug resistance and its short-term accumulation were common around this vulnerable time period. Results from this study, the first to examine HIV drug resistance specifically in the setting of community reentry, emphasize the vulnerability of this population to drug resistance and its potential subsequent treatment challenges. Moreover, the identification of some phylogenetically inferred transmission clusters, both within this JI population, and between it and the larger DC population, suggests the importance of including this population in molecular surveillance toward enhancement of HIV transmission prevention.

The high (43%–52%) overall DRM prevalence identified in this JI population exceeds previous U.S. reports among other JI populations in the United States, which have ranged from 16% to 43%,^4–7^ and is more similar to that from a recent analysis of a large, non-JI cohort in DC, which reported overall drug resistance of 45%.^29^ Additionally, the prevalence of DRMs to InSTIs in our study population was 9%–11%, which was higher than the 1.8% reported in the non-JI DC cohort study. We cannot make a direct comparison, but this finding may have been due to higher InSTI use in our cohort (26% of participants were taking an ART regimen that contained an InSTI) with suboptimal adherence. Our data suggest that recent incarceration may be associated with a high burden of ART resistance, possibly implying partial adherence, rather than full nonadherence, resulting in higher, rather than lower drug resistance. This increase in HIV drug resistance is likely multifactorial due to increased incidence of ART interruptions as persons cycle in and out of correctional facilities, poor ART adherence, substance use behaviors, unstable housing, lack of employment, and decreased access to health insurance.^30,31^

In this first study to evaluate the evolution of DRMs in the postincarceration period we demonstrated high (10–17 DRM in 21%–35% of participants) and rapid (0.12 new DRMs per person-months) accumulation of drug resistance, a rate even greater than two studies from South Africa that examined drug resistance accumulation in patients experiencing treatment failure on first-line ART.^32,33^ Conducting NGS, rather than conventional Sanger genotyping, allowed a more sensitive (5% rather than 20% threshold) evaluation of drug resistance and its accumulation in this vulnerable population.^34^ Though studies have demonstrated some clinical relevance of minority resistance variants detected between 5% and 20% NGS thresholds,^23,35^ this is still an ongoing research debate that mandates further investigation.^36^ Regardless, effective interventions are needed to address challenges during community reentry to improve and sustain viral suppression and decrease drug resistance emergence.

Several studies developed and tested interventions to improve HIV treatment outcomes among JI populations postrelease, with mixed results. In our CARE+ Corrections study, the mobile health intervention had a positive but nonsignificant association with viral suppression at 6 months, and care engagement increased in both the intervention and control groups.^37^ The SUCCESS trial, a strength-based case management intervention involving face-to-face sessions begun in jail and continuing postrelease, increased retention in care^38^; and the LINK LA trial, a peer navigation intervention that improved viral suppression at 12 months postrelease compared to traditional case management.^39^ In contrast, an intensive case management intervention in North Carolina among HIV-infected prisoners showed no improvement in retention in care over traditional prerelease discharge planning,^40^ demonstrating the need for further study of interventions to improve treatment outcomes during this vulnerable period. To date, there have been no prospective studies to examine the impact of such interventions on the emergence of drug resistance during community reentry. One potentially promising intervention to improve treatment adherence and viral suppression for high-risk patients includes the introduction of long-acting injectable ART medications.^41^ While these medications could improve adherence and viral suppression in JI populations during community reentry, they also pose new challenges such as the management of side effects, drug–drug interactions, and long-lasting drug concentrations that may further promote drug resistance.^42^

JI populations and correctional facilities may not typically be included in HIV surveillance programs, including molecular epidemiology and transmission networks inference. Despite having correctional health care systems and HIV treatment programs in many jurisdictions, these may be siloed and lack coordination with noncorrectional HIV public health programs. In addition, correctional health care providers may be less likely to pursue HIV drug resistance testing due to budgetary constraints. Our results, demonstrating clustering within this small JI cohort at relaxed criteria, and between this population and the DC community, demonstrate the significance of such inclusions. The lack of clustering with more stringent criteria for cluster definition suggests that clusters identified at the extended relaxed criteria likely represent historical, not recent, HIV transmissions. The same stringent criteria applied to the larger dataset identified 15 participants from the JI cohort in clusters with other non-JI individuals sampled in DC, which could suggest that these HIV transmissions did happen relatively recently, though this was not evaluated here. Interestingly, DRMs were not shared by cluster members implying that the observed drug resistance might be acquired and not transmitted. These data suggest that the JI populations are not truly sequestered from the broader community, but rather cycle between correctional facilities and the broader community and are at risk for HIV transmission given the risk of viremia. JI persons are an important component to understanding local HIV epidemics and transmission dynamics and efforts are needed to integrate them in surveillance and prevention efforts, particularly around community reentry.

This study had several limitations. First, the CARE+ Corrections study was not designed to evaluate drug resistance; therefore, this retrospective secondary analysis may not fully depict the prevalence and emergence of resistance in this population. Second, findings were limited by a relatively short follow-up period of 6 months and a small sample size with inadequate power to examine differences and associations. Third, HIV treatment status after the baseline assessment was not available, thus accumulation of drug resistance could not be confirmed to be the result of selective pressure from ART use. Lastly, NGS was performed without specific examination of recombination or quantification of input template (e.g., Primer ID),^43^ which limits accuracy of determining low minority resistance variants.

## Conclusions

In summary, this study identified high prevalence and accumulation of drug resistance in a JI population in DC during community reentry and demonstrated that this population was not isolated from the community HIV epidemic. This study sets the stage for prospective evaluation of prevalent and emergent HIV drug resistance in JI populations, intervention development to curb its emergence in this high priority population, and demonstrates the need for these JI populations to be included in broader surveillance and prevention efforts.

## Supplementary Material

Supplemental data
