# Supplementary material for: HIV Drug Resistance and Transmission Networks Among a Justice-Involved Population at the Time of Community Reentry in Washington, D.C
Source: AIDS Res Hum Retroviruses. 2021 Dec 15;37(12):903–12. doi: 10.1089/aid.2020.0267 (PMC8716515; doi:10.1089/aid.2020.0267)
Supplement: Supplemental data [file Supp_FigureS1.docx]

**

**

**Supplementary Figure S1.** Neighbor joining phylogenetic relationships among 54 study participants. The figure demonstrates a neighbor-joining phylogenetic sub-tree with the p-distance method of the same 54 study participants as Figure 4. The same three clusters identified in Figure 4 were identified here, and are highlighted in gray boxes. Bootstrap support ≥0.80 is shown at the node of each cluster and node size is proportional to bootstrap support. Other than one HIV-1 Subtype C sequence (the bottom sequence on the tree), all were HIV-1 subtype B (see Methods for HIV-1 subtyping details). The HIV-1 sequences that were used for tree rooting (3 HIV-1 subtype G sequences, accession numbers BE.96.DRCBL.AF084936, KE.93.HH8793-12-1.AF061641 and NG.92.92NG083.U88826; and 3 HIV-1 subtype H sequences, accession numbers BE.93.VI991.AF190127, BE.93.VI997.AF190128 and CF.90.056.AF005496) are not shown. The tree scale is shown at the bottom of the figure.
